# Neuroinflammation is responsible for pain in endometriosis - targeting the JAK-STAT pathway and mast cell activation

**DOI:** 10.3389/fimmu.2025.1621178

**Published:** 2025-08-29

**Authors:** Monika Golinska, Maria Wołyniak, Piotr Kulesza, Wojciech Fendler

**Affiliations:** ^1^ Department of Biostatistics and Translational Medicine, Medical University of Lodz, Lodz, Poland; ^2^ Cancer Research UK Cambridge Institute, University of Cambridge, Cambridge, United Kingdom; ^3^ Department of Radiation Oncology, Dana-Farber Cancer Institute, Boston, MA, United States; ^4^ Medical Research Agency, Warsaw, Poland

**Keywords:** endometriosis, neuroinflammation, chronic pain, Janus kinase inhibitors, mast cell stabilizers, neuromodulation

## Abstract

Chronic pain is a defining feature of endometriosis and contributes significantly to the diminished quality of life observed in affected individuals. Despite advances in understanding disease pathology, current therapeutic strategies largely fail to simultaneously target both lesion development and pain generation. In this review, we examine the neurobiology of endometriosis-associated pain at the level of the brain, dorsal root ganglia, and lesion innervation, with a particular focus on the interplay between inflammation and neurogenesis. We highlight how these processes converge on the JAK/STAT signaling pathway, a critical regulator of both immune activation and nerve fiber growth. The central role of mast cells in coordinating inflammatory and neurogenic responses is also discussed. Emerging evidence supporting the use of JAK inhibitors and mast cell stabilizers in modulating these pathways is reviewed, with emphasis on their potential for repurposing in endometriosis therapy. By targeting the shared mechanisms underlying lesion progression and pain, these pharmacological strategies offer a promising avenue for improving clinical outcomes. Further research is necessary to validate the efficacy and safety of these approaches, but the therapeutic potential of JAK/STAT pathway inhibition and mast cell stabilization could represent a paradigm shift in endometriosis management.

## 1 Introduction

Endometriosis is a chronic gynecological disorder characterized by chronic pain, excessive menstrual bleeding, and often infertility. The primary symptom, pelvic pain, typically intensifies during menstruation and may be accompanied by lower back and abdominal discomfort (1). Chronic pain associated with endometriosis is strongly linked to psychological distress and depression (2, 3), likely due to shared neural pathways in the amygdala, hypothalamus, and anterior cingulate gyrus- areas which regulate stress responses and emotional processing (4, 5). However, it remains unclear whether endometriosis directly amplifies pain perception or if individuals with the condition inherently exhibit heightened pain sensitivity, predisposing them to diagnosis. The mechanisms underlying pain perception in endometriosis are highly complex, as various gynecological conditions, including pregnancy, also contribute to pain hypersensitivity, the release of inflammatory mediators, and alterations in immune system function (6). Bajaj et al. (2) demonstrated on a group of woman diagnosed with endometriosis that nociceptive signaling from visceral endometriotic lesions triggers central sensitization, contributing to hyperalgesia. This process may play a key role in the persistence of pain or the recurrence of symptoms following medical or surgical treatment in women with symptomatic endometriosis. Similarly, in a murine model of endometriosis researchers observed heightened pain sensitization, along with increased anxiety and depressive behaviors (5).

Molecular events contributing to pain generation in endometriosis remain mostly unknown. A deeper understanding of these mechanisms is therefore critical to the development of targeted treatments that could address the underlying causes of chronic pain in endometriosis. In this review, we discuss how endometriosis microenvironment contributes to neurogenesis and pain signaling. We highlight the role of JAK/STAT pathway and mast cells activation in endometriosis-related inflammation. We also discuss the potential of application of JAK inhibitors and mast cells stabilizers in pain management in endometriosis.

### 1.1 The functional interplay between inflammation and neurogenesis in endometriosis

The relationship between inflammation and neurogenesis has been studied to some degree in other chronic pain conditions (7–10), but has not been well researched in endometriosis. Dysregulated immune system is believed to be a key driver of endometriosis contributing to inadequate removal of endometrium fragments from peritoneal cavity. This further stimulates local inflammation. Endometrial lesions contain increased amounts of activated peritoneal mast cells and macrophages and have a lowered cytotoxicity of T and NK cells (11–14). Higher levels of inflammatory mediators such as tumor necrosis factor α (TNFα), interleukins, prostaglandin E2 (PGE2), nerve growth factor (NGF) and chemokine RANTES/CCL5 were found in lesions and perioneal fluid of endometriosis patients (15, 16). It is likely that those factors also stimulate sensory nerve endings. Pelvic innervation was shown to differ in endometriosis sufferers compared to healthy controls. The presence of endometriosis-associated nerve fibers was correlated with pain perception in women affected by the condition (17). In those experiencing pelvic pain, the distance between endometrial glands and the nerve supply was found to be shorter (18). Additionally, nerve fiber densities were significantly higher in women with endometriosis compared to controls (19, 20). A greater number of nerve fibers were also identified in peritoneal endometriotic lesions than in normal peritoneum (21). Notably, subtypes of deep infiltrating endometriosis exhibited an even denser nerve supply compared to the innervation of the peritoneum (21).

**Figure 1:**
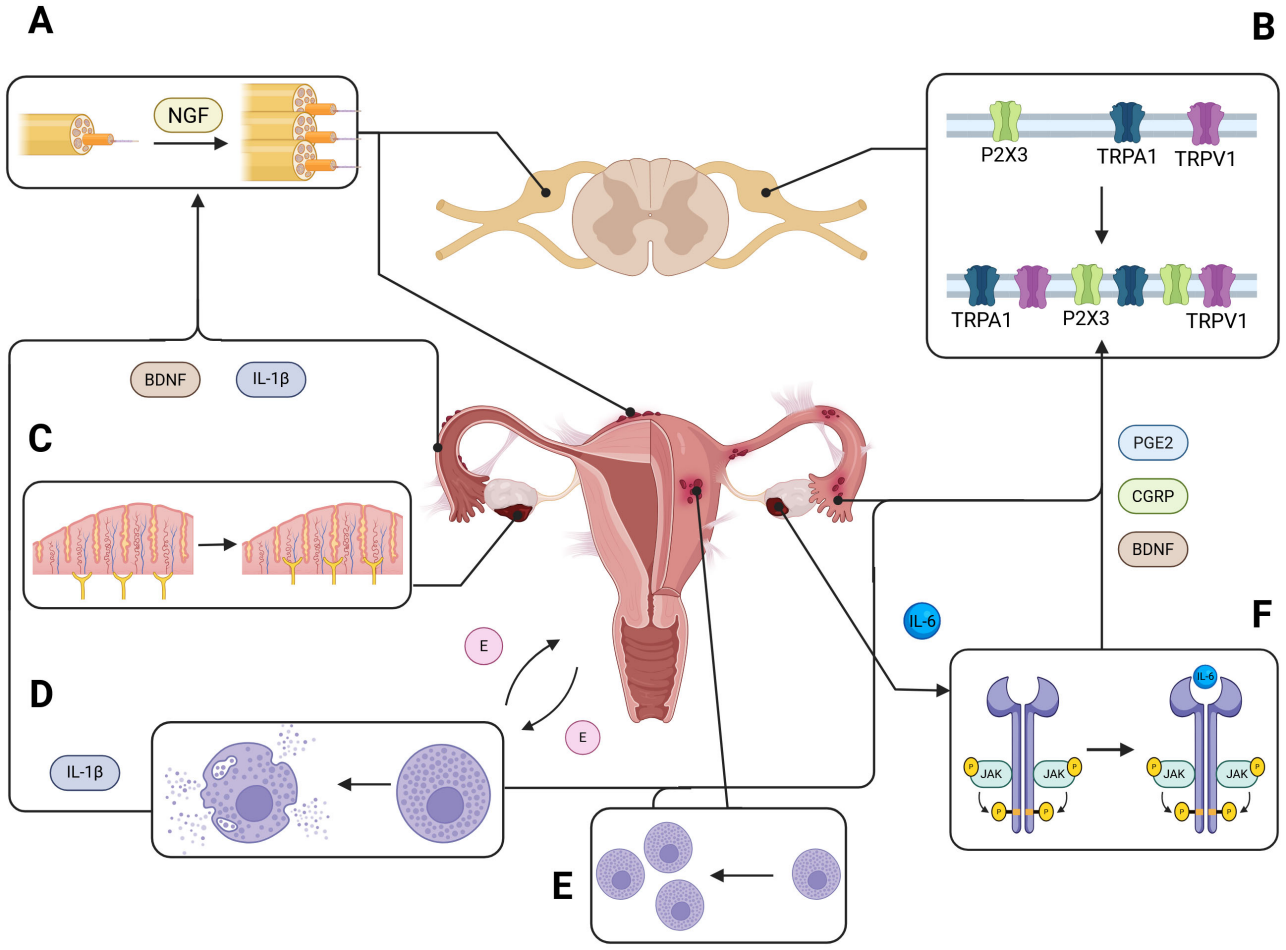
Neuroinflammation and peripheral nerve remodeling in endometriosis. This schematic illustrates the contribution of peritoneal inflammation to peripheral nerve growth and sensitization in endometriotic lesions. Endometrial lesions release a variety of inflammatory and neurotrophic mediators—including interleukin-1β (IL-1β), brain-derived neurotrophic factor (BDNF), and nerve growth factor (NGF)—that promote neurogenesis and the sprouting of sensory nerve fibers into ectopic tissue **(A)**. This aberrant innervation enhances nociceptive signaling to the dorsal root ganglia (DRG). Notably, elevated expression of transient receptor potential channels TRPV1 and TRPA1 has been observed in DRG neurons of individuals with endometriosis **(B)**, correlating with increased pain sensitivity and chronic pelvic pain. Within the peritoneal microenvironment, neurotrophic factors such as NGF and BDNF further drive excessive axonal sprouting and hyperinnervation of endometriotic lesions **(C)**. Endometrial tissue from both human and animal models shows increased infiltration and degranulation of mast cells **(D, E)**, which release inflammatory mediators that amplify local immune responses. Additionally, ectopic endometrial tissue produces cytokines like interleukin-6 (IL-6), which activate the JAK/STAT signaling pathway **(F)**, and upregulate the production of prostaglandin E2 (PGE2) and calcitonin gene-related peptide (CGRP), further exacerbating inflammation and pain.

It is possible that sensory nerves within endometriotic lesions interact with peritoneal immune cells by secreting neurotrophic factors. In peritoneal lesions, an increased number of macrophages have been observed to colocalize with nerve fibers (22), a process found to be driven by estradiol (23). Elevated immunoreactivity of NGF has also been reported near endometriotic glands (21). A recent study demonstrated that endometriosis-related pain is mediated through NGF-TrkA signaling (24). Furthermore, the estrogen-driven interplay between neurogenesis and the immune system has been shown to correlate with dysmenorrhea in endometriosis (25). The schematic of a cross-talk between inflammation and neurogenesis in endometriosis is presented in **Figure 1**.

### 1.2 Structural and biochemical brain changes in response to endometriosis generated pain

The brain, serving as the central coordination hub for all environmental stimuli, undergoes physiological transformations under their influence. Novel and intense stimuli activate neurons to restructure synaptic connections and form new ones, thereby initiating neurogenesis. Pain represents an exceptionally potent stimulus that induces significant modifications in the brain regions responsible for its processing. These changes are accompanied by observable electrophysiological alterations. In a study by Duric et al. (26) it was successfully demonstrated that pain can alter hippocampal morphology and gene expression in a mouse model of chronic and acute pain. Bromodeoxyuridine staining indicated that neurogenesis in the hippocampal dentate gyrus was significantly reduced after long-term inflammatory nociception, implicating that prolonged nociceptive input triggers maladaptive neural plasticity, including the remodeling of synaptic connections and disruption of neurogenic processes. These findings suggest that neural changes induced by chronic pain, such as impaired neurogenesis and maladaptive plasticity, may play a significant pathophysiological role in endometriosis-associated pain.

Mice carrying endometriosis exhibited a higher sensitivity to pain, and showed signs of depression and anxiety. Their insula, amygdala, hippocampus and cerebral cortex all showed differential gene expression compared to non-endometriosis controls (27). The central nucleus of the amygdala, a region critically involved in pain perception and emotional processing, revealed significant alterations in gene expression and intrinsic electrophysiological properties of neurons in murine model of endometriosis (5). Others have reported an increased microglial soma size in the cortex, hippocampus, thalamus, and hypothalamus of mice with endometriosis (28). Together, these observations underscore the contribution of central nervous system changes—such as neural remodeling, neuroinflammation, and sensitization processes—to the development and maintenance of chronic pain in endometriosis.

**Figure 2:**
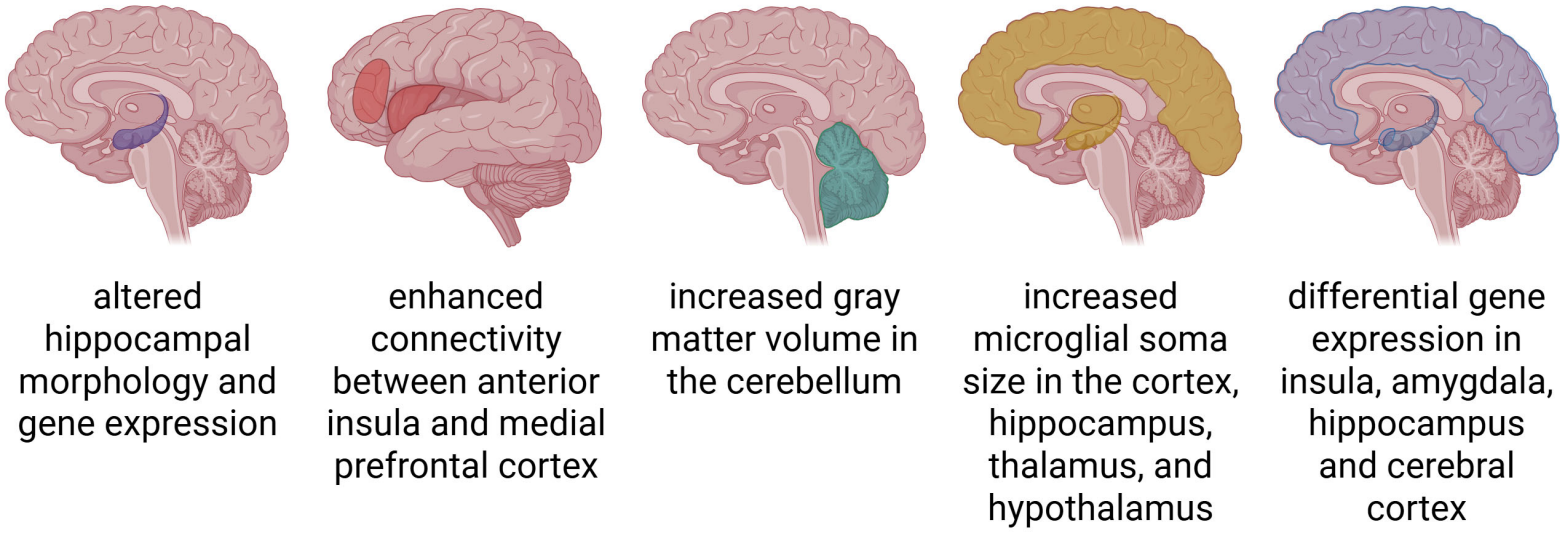
Structural and functional adaptations in the brain associated with chronic pain. Chronic pain leads to widespread neuroplastic changes across the brain. These include altered brain morphology, such as increases in grey matter volume or microglial soma size; enhanced functional and structural connectivity between pain-processing regions; and differential gene expression in key areas such as the hippocampus, insula, amygdala, and cerebral cortex.

Women with endometriosis exhibit distinct neurobiological patterns compared to other patients with chronic pelvic pain. They show increased gray matter volume in the cerebellum (29). In addition, compared to pain-free controls, women with endometriosis demonstrate enhanced connectivity between the anterior insula and the medial prefrontal cortex, two regions heavily involved in pain perception and processing. Furthermore, proton magnetic resonance spectroscopy revealed altered brain chemistry in these patients, with higher concentrations of combined glutamine and glutamate in the anterior insula (30). Adaptations in the brain in response to chronic pain are presented in **Figure 2**.

### 1.3 Dorsal root ganglion and its role in chronic pain in endometriosis

Dorsal root ganglion (DRG) neurons play a central role in mediating chronic pain associated with endometriosis. Acting as key relays between peripheral tissues and the central nervous system, DRG neurons undergo significant structural and molecular changes, driven by neurotrophic factors, such as NGF and brain-derived neurotrophic factor (BDNF). These factors have been documented in both human tissue samples and rodent models of endometriosis (31, 32) and they were found to promote excessive axonal sprouting and hyperinnervation of endometriotic lesions, amplifying nociceptive input to the spinal cord (33) (**Figure 1A**). Additionally, BDNF and calcitonin gene-related peptide (CGRP) contribute to pain amplification by enhancing neuronal excitability and fostering neuroimmune interactions within the DRG (31, 34).

Recent research has highlighted the molecular mechanisms underlying DRG neuron hyperexcitability in endometriosis, with a focus on ion channel regulation (see **Figure 1B**). Transient receptor potential (TRP) channels, particularly TRPV1 and TRPA1, have emerged as key mediators of nociception in sensory neurons. Elevated expression of TRPV1 and TRPA1 has been observed in both human and rodent DRG neurons, correlating with heightened pain sensitivity and estrogen regulation (35–37). The pharmacological blockade of TRPV1 has demonstrated effectiveness in reducing pain responses in animal models, reinforcing its role in peripheral sensitization (35). In addition to TRP channels, sodium channels, such as NaV1.7, are significantly upregulated in neurons innervating pelvic organs affected by endometriosis. Functional studies have shown that NaV1.7 activation increases nociceptive sensitivity, further contributing to pain amplification (38). Purinergic signaling via the P2X3 receptor is another critical mechanism in pain modulation. Upregulation of P2X3 in DRG neurons has been linked to activation of the ATF3/AP-1 and ERK signaling pathways in animal models of endometriosis, establishing its role as a driver of nociceptive signaling (39–41). These findings underscore the central role of ion channel upregulation in DRG neuron hyperexcitability and its contribution to pain sensitization in endometriosis. Emerging therapeutic strategies have targeted these pathways. Experimental models have shown that CGRP-RAMP1 inhibition reduces endometriotic lesion size and pain responses, in mice suggesting clinical applications for modulating DRG-mediated nociceptive signaling (42). Moreover, research into DRG neuromodulation highlights its potential as a promising strategy for treating chronic pain, given the unique physiology and accessibility of DRG neurons (35–37). These findings underscore the interconnected role of neuroimmune and inflammatory signaling in peripheral sensitization and chronic pain management.

### 1.4 Changes in structure and location of neurons in endometriosis

Endometriosis is characterized by the aberrant presence of pathological neurons within affected tissues (43) (**Figure 1C**). NGF and neurotrophin-3 (NT-3), are expressed in peritoneal endometriotic implants and in the peritoneal fluid of patients with endometriosis (44). NGF plays role in inflammation and pain processing: inflammatory cells stimulate the release of neuroactive cytokines and inflammatory mediators, with IL-1β and TNF-α acting as potent triggers for NGF synthesis (45) (see **Figure 1A**). Advances in immunohistochemistry have facilitated the detailed analysis of neuronal marker distribution in endometrial tissue, the myometrium, and endometriotic lesions, enabling a more precise understanding of the neural components involved in the disease pathology. In their research, Al-Jefout et al. (46) reported that the distance between endometrial glands and nerve fibers in endometriotic lesions was significantly shortened in women experiencing pain compared to those without pain (assessed with pain score). Additionally, women with endometriosis and pain symptoms exhibited a significantly higher density of nerve fibers than women with endometriosis- related infertility but no pain (46). Arnold et al. (47) identified an imbalance between sympathetic and sensory nerve fibers in peritoneal endometriosis. The peritoneal fluid of patients with endometriosis, compared to that from women without the disease, was shown to induce increased sprouting of sensory neurites from dorsal root ganglia while simultaneously inhibiting neurite outgrowth from sympathetic ganglia. Sensory C and autonomic nerve fibers were observed in the functional layer of the endometrium exclusively in women with endometriosis, with even greater densities detected in the basal layer, which implicates the possible role in maintenance of inflammation and pain (47). Tokushige et al. (19) further described the presence of numerous small unmyelinated nerve fibers in the functional layer of the endometrium in patients with endometriosis. These nerve fibers were predominantly located in the deeper portion of the functional layer, though some were observed in close proximity to the endometrial epithelial surface, particularly near blood vessels and glands. In contrast, no nerve fibers were detected in the functional layer of the endometrium in patients without endometriosis (21). Interestingly, the density of nerve fibers was greater during the secretory than in the menstrual or proliferative phases of the endometrial cycle (21).

### 1.5 Mast cells in inflammation, pain and endometriosis

Mast cells play a key role in neuromodulation by mediating a crosstalk between immune and nervous system (48, 49). They release a range of biomediators that impact neuroinflammation and pain signaling. The proliferation and homeostasis of mast cells is dependent on JAK/STAT pathway (50). The primary physiologic function of mast cells is to facilitate immune responses to infectious agents (51) but they are also involved in the degradation of toxic endogenous peptides (52) and wound healing responses (53–55). Overactivated mast cells are best known for promoting chronic inflammatory disorders and are early effectors of allergic disease, urticaria, anaphylaxis, arthritis, and asthma (56, 57). Their dysfunction can lead to mast cell activation syndrome and they have been shown to contribute to ulcerative colitis, Crohn’s disease and multiple sclerosis among others (58, 59). Mast cells can cause profound inflammation and vasodilation and release inflammatory mediators including histamines, cytokines (TNF, IL-1, IL-6, IL-8), tryptase, chymase, prostaglandins and growth factors (60, 61).

Mast cells are crucial in the development of protective pain and they are thought to contribute to the regulation of pain signaling (62, 63). The role of mast cells has been correlated with the development of chronic pain in various conditions including rheumatoid arthritis, irritable bowel syndrome, interstitial cystitis, chronic pelvic pain syndrome and chronic prostatitis and in several brain disorders (63, 64). Activated mast cells are likely to sensitize primary nociceptive neurons by the release of histamines, NGF and pro-inflammatory cytokines TNF and IL-8; the latter has been shown to activate prostaglandin-independent hyperalgesia *in vivo* (65).

Mast cells are speculated to play a key role in endometriosis; they impact the recruitment and performance of other immune cells. Increased levels of mast cells and their augmented degranulation was shown in endometrial tissue of animal models and humans (66–68) (**Figures 1D, E**). We have shown that endometrial lesion tissue has an increased expression of mast cell markers including KIT, CPA3 and both MS4A2 and MS4A6A compared to control endometrium (69). Mast cells were found to promote endometrial cells migration in *in vitro* assays (70) and shown to colocalize to the vasculature of ovarian endometriomas. Endometrial lesions secrete mediators such as nerve growth factor (NGF), vascular endothelial growth factor (VEGF), and substance P, which are known to attract mast cells. Studies have shown that increased mast cell infiltration and degranulation are influenced by NGF production within endometrial tissue (33). Mast cells are believed to partake in neuropathic pain development in endometriosis. Pain intensity in patients correlated positively with an elevated number of mast cells in lesions, which were mostly located near nerve fibers (71).

Human MrgprX2 and its murine ortholog MrgprB2 are G protein–coupled receptors selectively expressed on connective-tissue mast cells. These receptors mediate rapid, non-IgE-dependent mast cell activation in response to neuropeptides such as substance P and PACAP, as well as various drugs and cationic peptides. Their activation establishes a bidirectional communication axis between mast cells and peripheral sensory neurons, promoting neurogenic inflammation and pain (72, 73).

*In vivo* studies across multiple tissues-including skin, meninges, bladder, and gut-have demonstrated the functional importance of this pathway. In murine models of interstitial cystitis/bladder pain syndrome (IC/BPS), MrgprB2-dependent mast cell–neuron circuits promote bladder inflammation and colonic hypersensitivity; MRGPRX2 antagonism reduces these effects in humanized mice (74). Similarly, in migraine models, MrgprB2+ meningeal mast cells respond to compound 48/80 or PACAP with pain behaviors absent in knockout animals; MRGPRX2+ humanized mice show enhanced responses (72). In the skin, MrgprB2 mediates mast cell degranulation and neurogenic inflammation induced by substance P, LL-37, and drugs like compound 48/80, with effects abolished by receptor knockout or antagonism (73). In the gut, human colonic mast cells express MRGPRX2 and respond to agonists, with altered profiles in IBS biopsies (75).

Despite this broad relevance, MrgprX2/B2 signaling has not been studied in gynecological tissues to-date. The presence of MrgprB2+ mast cells was shown in the uterus and mammary gland (76), yet no *in vivo* data addresses receptor activation, neuron interaction, or its roles in pain or lesion formation.

This gap limits our understanding of mast cell–neuron signaling in reproductive disorders. Future studies could reveal whether MrgprX2/B2 contributes to pelvic pain and inflammation in conditions like endometriosis offering new targets for non-hormonal therapy.

### 1.6 Role of JAK/STAT pathway in endometriosis and pain development

Both neurogenesis and inflammation converge on JAK/STAT pathway, which provides a rapid membrane-to-nucleus signaling platform that mediates cellular inflammation response (77, 78). This central pathway is responsible for transmitting extracellular signals that govern migratory and invasive properties of cells. JAK/STAT pathway overactivation was shown to facilitate proliferation and angiogenesis in cancer (79), in breast malignancies its prolonged activation was linked with tumor development (80) and therapy resistance (81).

Various cytokines that play a role in the development of inflammatory and autoimmune diseases utilize JAKs and STATs proteins to transmit intracellular signals and impact homeostasis. Dysregulation of JAK/STAT pathway and mutations and polymorphisms in its genes correlate with numerous immunodeficiency syndromes (82, 83). Altered JAK/STAT signaling was detected in endometriosis (84) (**Figure 1F**). Phosphorylation of STAT3 was upregulated in endometriosis lesions (85) and activated STAT3 increased proliferation of endometrial stromal cells (86). IL-6 mediated activation of STAT3 was correlated with fibrosis in endometriosis (87).

The JAK/STAT pathway plays an essential role in maintenance of neural stem cells (NSCs) (88, 89) and pain signaling (90). On the other hand, cytokine IL-15 produced by adult NSCs contributes to STAT proteins activation (91). STAT3 has been shown to regulate neuropathic pain maintenance in rats. Pain resulting from nerve injury was accompanied by an increase in STAT3 phosphorylation in microglial cells of dorsal spinal cord (92) and by an increase in JAK/STAT signaling and resulting augmented proliferation of astrocytes (93). IL-6 signaling system is known to activate JAK/STAT pathway; increased expression of IL-6 mRNA was observed in dorsal root ganglia and in dorsal spinal cord of rats with neuropathic pain (92). JAK/STAT pathway was shown to be involved in synaptic plasticity in the brain (94).

Gain-of-function mutations in the JAK–STAT signaling pathway are well-established molecular drivers of immune dysregulation (95–98). Such mutations typically enhance cytokine responsiveness or lead to constitutive activation of downstream transcriptional programs, often by disrupting autoinhibitory domains or promoting ligand-independent phosphorylation and dimerization (95, 98). Those gain-of-function mutations have been found in various auto-immune diseases.

JAK1 mutations are associated with severe allergic disease, eosinophilia, and Th2-biased inflammation (95, 99). STAT3 gain-of-function mutations lead to autoimmunity, lymphoproliferation, and hypogammaglobulinemia. Mechanistic studies using overexpression systems, CRISPR murine models, and phospho-signaling assays have confirmed their pathogenicity and responsiveness to JAK inhibitors (98, 100). STAT6 gain-of-function mutations increase basal activity and IL-4/IL-13 sensitivity, promoting allergic phenotypes and, in some cases, lymphoma (96, 97).

To date, no gain-of-function mutations in JAK1, STAT3, STAT5B, or STAT6 have been identified in human endometriosis tissues, cell lines, or animal models. This remains an important area for future investigation. Introducing validated gain-of-function alleles into endometrial stromal or epithelial models could provide a powerful strategy to uncover causal mechanisms and potentially reveal novel therapeutic targets.

The JAK/STAT pathway varies by tissue context. In the eutopic endometrium, cyclical fluctuations in macrophages, neutrophils, and uterine natural killer (uNK) cells play a vital role in tissue remodeling and immune regulation (101). In endometriosis, however, these immune cell populations—particularly macrophages and neutrophils—are dysregulated, especially during the proliferative phase, suggesting a disruption in immune–neural communication (102). The JAK/STAT pathway is a key regulator of immune cell function, including the pain-related activity of macrophages. Dysregulation of this pathway—particularly through IL-23/IL-17 signaling and the TYK2-JAK2 axis—can enhance neutrophil activation and contribute to pain sensitization. Pharmacological inhibition of this pathway has been shown to reduce inflammatory responses in other autoimmune diseases and is therefore likely to be beneficial in endometriosis.

### 1.7 Translational potential of repurposing JAK/STAT inhibitors and mast cell stabilizers for endometriosis

#### 1.7.1 Current research and clinical use of JAK inhibitors in autoimmune diseases

Various JAK inhibitors are approved for the treatment of autoimmune and inflammatory diseases and some for the treatment of myeloproliferative neoplasms (103, 104); others are considered for treatment of solid tumors (105). JAK inhibitors have been reviewed extensively elsewhere (104, 106, 107). Those approved for clinical use are summarized in **Table 1**. In this review, we discuss those applications that could be relevant in relieving endometriosis symptoms. Tofacitinib, JAK inhibitor, has emerged as a key therapeutic agent for managing autoimmune conditions such as rheumatoid arthritis, psoriatic arthritis, dermatitis, and ulcerative colitis (110). By inhibiting the phosphorylation of JAK enzymes, tofacitinib blocks their activation within the JAK/STAT pathway, which is critical for initiating and sustaining inflammatory responses. It was shown that the inhibition of suppressor of cytokine signaling 3 (SOCS3)—an inducible negative regulator of the JAK/STAT pathway that is strongly expressed in the adipose tissue of patients with Graves’ orbitopathy—resulted in a significant reduction in both proinflammatory cytokine production and adipogenesis (111). Gupta et al. (112) showed that targeting the JAK-STAT pathway alleviates salivary gland inflammation and mitigates interferon-mediated immune activation in Sjögren’s disease (112). Moreover, JAK inhibitors were shown to be effective and safe in the treatment of refractory, moderate-to-severe Crohn’s disease (CD) and ulcerative colitis (UC) (107). As a result, they are utilized as second-line therapeutic agents in the management of inflammatory bowel disease (IBD), particularly in patients who have an inadequate response or intolerance to conventional therapies such as corticosteroids, immunomodulators, or biologic agents. The targeted modulation of the JAK/STAT signaling pathway, allows for the attenuation of proinflammatory cytokine signaling, which plays a central role in the pathogenesis of IBD.

**Table 1:** JAK inhibitors used in clinic (108, 109).

JAK inhibitor	Target	Disease
Tofacitinib	JAK 1,2,3	Rheumatoid arthritis, ulcerative colitis
Ruxolitinib	JAK 1,2	myelofibrosis, polycythemia vera
Baricitinib	JAK 1,2	Alopecia areata, atopic dermatitis, rheumatoid arthritis
Peficitinib	JAK 1,2,3	Rheumatoid arthritis
Upadacitinib	JAK 1,2,3	Atopic dermatitis, ankylosing spondylitis, psoriatic arthritis, rheumatoid arthritis, ulcerative colitis,
Delgocitinib	JAK 1,2,3	Atopic dermatitis
Filgotynib	JAK 1,2,3	Rheumatoid arthritis, ulcerative colitis
Abrocitinib	JAK 1,2	Atopic dermatitis

The JAK/STAT signaling pathway has been identified as a key mechanism in the development and pathophysiology of inflammatory joint diseases. Yang et al. (113) demonstrated that matrine may exert therapeutic effects in collagen-induced arthritis by suppressing the proliferation of fibroblast-like synoviocytes and promoting their apoptosis, at least in part through the downregulation of the JAK/STAT signaling cascade (113). Furthermore, activation of the JAK/STAT pathway by interferon-γ has been shown to confer resistance to apoptosis in synovial cells in the context of inflammatory rheumatoid arthritis, contributing to their excessive proliferation (106). In a study by Suda et al. (114), the inhibitory effects of five JAK inhibitors—including tofacitinib, baricitinib, peficitinib, upadacitinib, and filgotinib—were compared with respect to interleukin (IL)-6-induced inflammation in rheumatoid arthritis (RA) synovial tissues. The findings indicate that although all five JAK inhibitors were shown to reduce IL-6-induced inflammatory and angiogenic factors, variations in their efficacy may be attributed to distinct molecular mechanisms and pharmacological characteristic (114).

#### 1.7.2 Role of mast cells stabilizers in autoimmune diseases

Mast cell stabilizers mitigate allergic responses by inhibiting mast cell degranulation and preventing the release of vasoactive mediators, including histamine. The stabilizers approved for clinical use are summarized in **Table 2**. Owing to their mechanism of action, mast cell stabilizers represent a promising class of therapeutic agents for the treatment of autoimmune and inflammatory diseases characterized by eosinophilic infiltration and mast cell degranulation. Conditions such as asthma and eosinophilic esophagitis, in which these immunological processes play a central role, are thought to particularly benefit from the ability of mast cell stabilizers to inhibit the release of pro-inflammatory mediators, thereby reducing tissue inflammation and associated symptoms. Examples of drugs in this group include among others: Cromolyn sodium, Nedocromil sodium and Lodoxamide trometamol. Although their precise mechanism of action remains unclear, these compounds are believed to exert their effects by stabilizing the mast cell membrane and restricting calcium influx (115, 116). Despite structural diversity within this class of agents, their shared ability to modulate calcium dynamics is thought to be central to their function. Lodoxamide, a mast cell stabilizer was assessed for its effectiveness in reducing local allergic responses in the conjunctiva of rats through *in vivo* testing, as well as its capacity to suppress the release of mediators from rat conjunctival mast cells *in vitro* (117). The findings indicate that lodoxamide’s anti-allergic effects observed in the conjunctiva are closely linked to its ability to inhibit the release of allergic mediators from mast cells located within this tissue (117). Given the inflammatory role of mast cells and their presence in the CNS, the effect of ketotifen fumarate, a mast cell stabilizer, was investigated in the context of encephalomyelitis development in mouse model. A significant reduction in disease severity and prevalence was observed following early ketotifen treatment. This protective effect was associated with reduced NLRP3 inflammasome activation, rebalanced oxidative stress, and decreased T cell infiltration in the CNS. These findings reinforce the relevance of mast cells in encephalomyelitic spathogenesis and suggest that Ketotifen may serve as a potential therapeutic approach (116) Evidence from randomized controlled trials indicates that ketotifen, either used alone or in combination with other interventions, improves asthma control and reduces wheezing in children with mild to moderate asthma. These findings suggest that ketotifen may be an effective therapeutic option in pediatric asthma management, particularly in cases where inflammation and allergic responses play a significant role (118) To the present day there are no clinical trials considering mast cell stabilizers in endometriosis, but Li-bo Zhu et al. (119) revealed that sodium cromoglycate can stabilize mast cells from degranulation, thus relieving the clinical endometriosis symptoms in a rat model.

**Table 2:** Mast cells stabilizers approved for clinical use (120–122).

Mast cell stabilizer	Target	Disease
Cromolyn Sodium	Mast cells degranulation inhibition	Allergic rhinitis, asthma, mastocytosis, and certain allergic conjuctivitis
Ketotifen	Blockage of H1 receptors	Allergic rhinitis, asthma, atopic dermatitis, conjuctivitis
Olopatadine	Blockage of H1 receptors	Conjuctivitis
Lodoxamide	Mast cells degranulation inhibition	Vernal keratoconjuctivitis
Nedocromil	Mast cells degranulation inhibition	Mild to moderate asthma
Pemirolast	Decreased histamine release	Allergic conjuctivitis

## 2 Discussion

Chronic pain is a hallmark of endometriosis, yet effective strategies for pain management remain limited. Many patients ultimately require surgical removal of lesions, and even this approach does not resolve all pelvic pain problems. There is an urgent need to better understand the mechanisms underlying pain generation and signaling in endometriosis to guide the development of targeted therapies.

In this review, we present evidence from animal models demonstrating that structural and molecular alterations in key brain regions involved in pain perception—such as the amygdala, hippocampus, and cerebral cortex—are most likely linked to increased pain sensitivity and the persistence of pain, which in turn contributes to emotional disturbances such as anxiety and depression. We also highlight that pain-related changes in endometriosis occur at multiple levels, ranging from structural brain alterations, through changes in the dorsal root ganglia, to increased neuronal density in close proximity to endometriotic lesions. These observations underscore the need for therapeutic strategies that address chronic pain in endometriosis across these various levels. In this context, we propose that preventing mast cell degranulation, along with targeting the JAK-STAT signaling pathway—particularly through its inhibition—may offer a promising therapeutic strategy for effective intervention.

**Figure 3:**
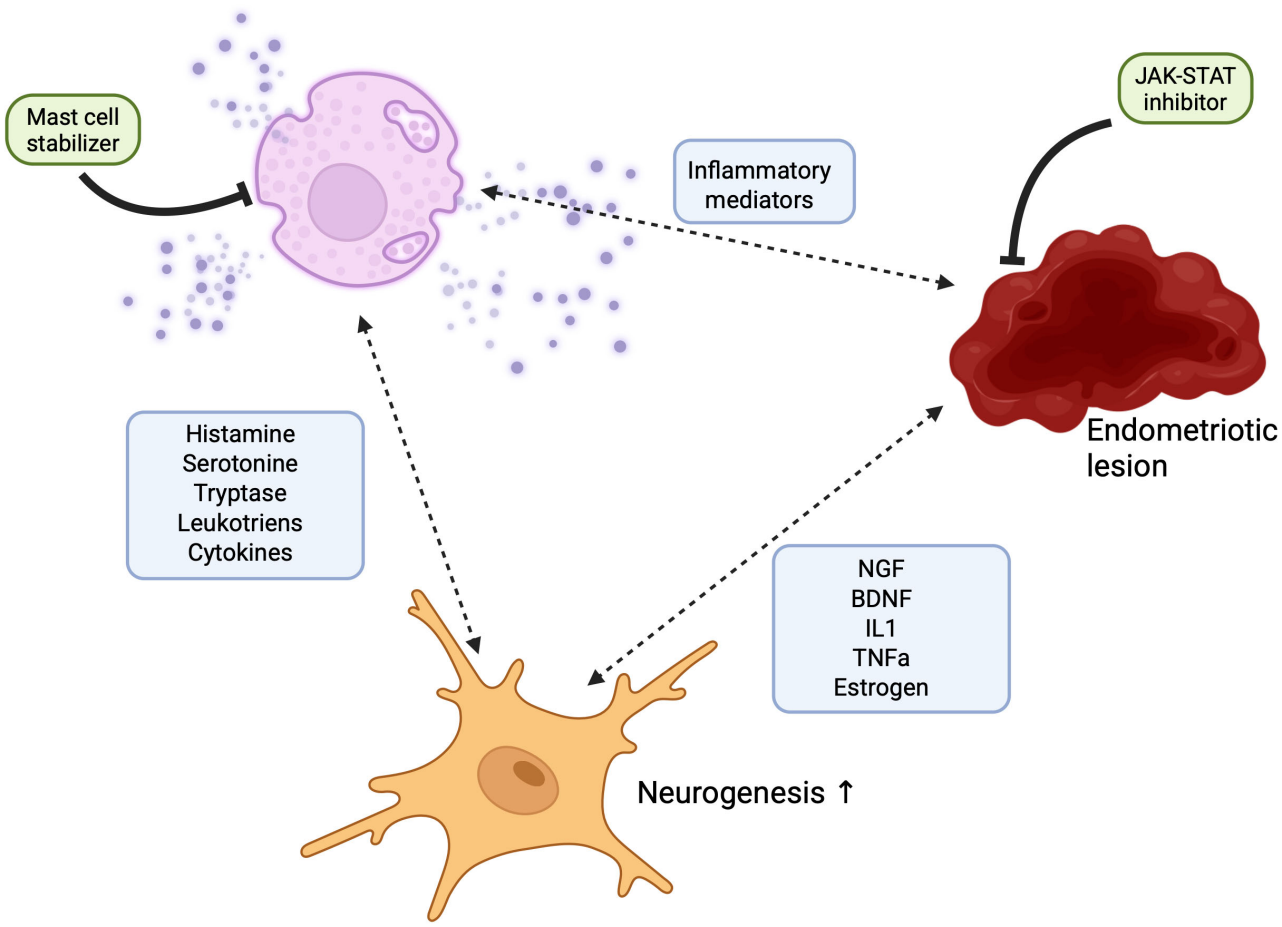
Schematic representation of the neuro-immune loop in endometriosis. This figure illustrates the proposed interaction between endometrial lesions, peripheral neurons, and mast cells, contributing to chronic inflammation and pain in endometriosis. Ectopic endometrial lesions release pro-inflammatory cytokines and neurotrophic factors, which activate nearby mast cells and promote neuronal sensitization. Activated mast cells degranulate, releasing inflammatory mediators such as histamine, tryptase, and prostaglandins, which further sensitize neurons and sustain the inflammatory environment. This establishes a self-amplifying neuro-immune loop. The schematic also highlights the potential therapeutic effects of Janus kinase (JAK) inhibitors and mast cell stabilizers, which may disrupt this loop by reducing the release of inflammatory mediators.

The JAK/STAT signaling pathway plays a pivotal role in regulating inflammatory responses and is essential for proper mast cell function. Notably, the JAK/STAT axis has emerged as a critical mediator of neuroinflammation, making it a compelling target for investigating the pathophysiology of endometriosis-associated chronic pain. In this review, we examine the mechanisms underlying pain signaling in endometriosis and their relationship to lesion development, highlighting how key mediators involved in JAK/STAT signaling and mast cell activation contribute to pain generation. We present emerging evidence that underscores the significance of the JAK/STAT pathway in endometriosis-related neuroinflammation and emphasize the central role of mast cells in modulating pain. We propose that these two components—JAK/STAT signaling and mast cell activity—require deeper investigation, particularly regarding their potential interaction in the context of chronic pain in endometriosis (see **Figure 3**).

A recent case report described a 38-year-old woman with rheumatoid arthritis and primary infertility who conceived six months after initiating JAK inhibitor therapy with tofacitinib. Although there are no previous reports linking tofacitinib to improved fertility in cases related to endometriosis, this finding raises the possibility of a connection between JAK pathway inhibition, inflammation associated with endometriosis, and fertility outcomes. Similarly, such association should be explored in relation to endometriosis-associated pain. Further well-designed studies are needed to investigate the therapeutic potential and safety of JAK inhibitors in the treatment of endometriosis.

To date, no comprehensive study has examined the interplay between inflammation, neurogenesis, mast cell activation, and JAK/STAT signaling in endometriosis. Addressing this gap is crucial, as these pathways are increasingly recognized as key contributors to disease progression and pain. Although there is currently no direct evidence supporting the efficacy of JAK inhibitors or mast cell stabilizers in endometriosis, the well-documented overactivation of both pathways in the disease highlights their potential as therapeutic targets. Drawing on evidence from other autoimmune and chronic pain conditions—as well as current insights into JAK/STAT signaling and mast cell involvement in endometriosis—we propose that evaluating these agents represents a promising and necessary direction for future research and therapeutic development.

## References

[1] Mitchell AM Lensen S Kamper SJ Frawley H Cheng C Healey M. The most impactful endometriosis symptom: An international, cross-sectional, two-round survey study. Acta Obstet Gynecol Scand. (2024) 103:1736–44. doi: 10.1111/aogs.14927, PMID: 39041353 PMC11324914

[2] Kalfas M Chisari C Windgassen S. Psychosocial factors associated with pain and health-related quality of life in Endometriosis: A systematic review. Eur J Pain. (2022) 26:1827. doi: 10.1002/ejp.2006, PMID: 35802060 PMC9543695

[3] Gambadauro P Carli V Hadlaczky G. Depressive symptoms among women with endometriosis: a systematic review and meta-analysis. Am J Obstet Gynecol. (2019) 220:230–41. doi: 10.1016/j.ajog.2018.11.123, PMID: 30419199

[4] Zheng P Zhang W Leng J Lang J. Research on central sensitization of endometriosis-associated pain: a systematic review of the literature. J Pain Res. (2019) 12:1447. doi: 10.2147/JPR.S197667, PMID: 31190954 PMC6514255

[5] Li T Mamillapalli R Ding S Chang H Liu ZW Gao XB. Endometriosis alters brain electrophysiology, gene expression and increases pain sensitization, anxiety, and depression in female mice. Biol Reprod. (2018) 99:349–59. doi: 10.1093/biolre/ioy035, PMID: 29425272 PMC6692844

[6] Bajaj P Bajaj P Madsen H Møller M Arendt-Nielsen L. Antenatal women with or without pelvic pain can be characterized by generalized or segmental hypoalgesia in late pregnancy. J Pain. (2002) 3:451–60. doi: 10.1054/jpai.2002.128065, PMID: 14622731

[7] Bernardino L Agasse F Malva JO. (2009). Neurogenesis and Inflammation. In: Binder, M.D., Hirokawa, N., Windhorst, U. (eds) Encyclopedia of Neuroscience. Springer, Berlin, Heidelberg. doi: 10.1007/978-3-540-29678-2_3847

[8] Chen L Qin Q Huang P Cao F Yin M Xie Y. Chronic pain accelerates cognitive impairment by reducing hippocampal neurogenesis may via CCL2/CCR2 signaling in APP/PS1 mice. Brain Res Bull. (2023) 205:110801. doi: 10.1016/j.brainresbull.2023.110801, PMID: 37931808

[9] Grilli M. Chronic pain and adult hippocampal neurogenesis: translational implications from preclinical studies. J Pain Res. (2017) 10:2281. doi: 10.2147/JPR.S146399, PMID: 29033604 PMC5614764

[10] Tyrtyshnaia A Manzhulo I Kipryushina Y Ermolenko E. Neuroinflammation and adult hippocampal neurogenesis in neuropathic pain and alkyl glycerol ethers treatment in aged mice. Int J Mol Med. (2019) 43:2153–63. doi: 10.3892/ijmm.2019.4142, PMID: 30896810 PMC6445594

[11] Garzetti GG Ciavattini A Provinciali M Muzzioli M Di Stefano G Fabris N. Natural killer activity in stage III and IV endometriosis: impaired cytotoxicity and retained lymphokine responsiveness of natural killer cells. Gynecol Endocrinol. (1995) 9:125–30. doi: 10.3109/09513599509160201, PMID: 7502688

[12] Harada T Enatsu A Mitsunari M Nagano Y Ito M Tsudo T. Role of cytokines in progression of endometriosis. Gynecol Obstet Invest. (1999) 47 Suppl 1:34–40. doi: 10.1159/000052857, PMID: 10087426

[13] Jones RK Bulmer JN Searle RF. Phenotypic and functional studies of leukocytes in human endometrium and endometriosis. Hum Reprod Update. (1998) 4:702–9. doi: 10.1093/humupd/4.5.702, PMID: 10027623

[14] Szyllo K Tchorzewski H Banasik M Glowacka E Lewkowicz P Kamer-Bartosinska A. The involvement of T lymphocytes in the pathogenesis of endometriotic tissues overgrowth in women with endometriosis. Mediators Inflammation. (2003) 12:131–8. doi: 10.1080/0962935031000134842, PMID: 12857596 PMC1781609

[15] Cheong YC Shelton JB Laird SM Richmond M Kudesia G Li TC. IL-1, IL-6 and TNF-alpha concentrations in the peritoneal fluid of women with pelvic adhesions. Hum Reprod. (2002) 17:69–75. doi: 10.1093/humrep/17.1.69, PMID: 11756364

[16] Khorram O Taylor RN Ryan IP Schall TJ Landers DV. Peritoneal fluid concentrations of the cytokine RANTES correlate with the severity of endometriosis. Am J Obstet Gynecol. (1993) 169:1545–9. doi: 10.1016/0002-9378(93)90433-J, PMID: 7505529

[17] McKinnon B Bersinger NA Wotzkow C Mueller MD. Endometriosis-associated nerve fibers, peritoneal fluid cytokine concentrations, and pain in endometriotic lesions from different locations. Fertil Steril. (2012) 97:373–80. doi: 10.1016/j.fertnstert.2011.11.011, PMID: 22154765

[18] Tulandi T Felemban A Chen MF. Nerve fibers and histopathology of endometriosis-harboring peritoneum. J Am Assoc Gynecol Laparosc. (2001) 8:95–8. doi: 10.1016/S1074-3804(05)60556-7, PMID: 11172122

[19] Tokushige N Markham R Russell P Fraser IS. High density of small nerve fibres in the functional layer of the endometrium in women with endometriosis. Hum Reprod. (2006) 21:782–7. doi: 10.1093/humrep/dei368, PMID: 16253968

[20] Tokushige N Markham R Russell P Fraser IS. Different types of small nerve fibers in eutopic endometrium and myometrium in women with endometriosis. Fertil Steril. (2007) 88:795–803. doi: 10.1016/j.fertnstert.2006.12.078, PMID: 17451690

[21] Tokushige N Markham R Russell P Fraser IS. Nerve fibres in peritoneal endometriosis. Hum Reprod. (2006) 21:3001–7. doi: 10.1093/humrep/del260, PMID: 16950827

[22] Tran LVP Tokushige N Berbic M Markham R Fraser IS. Macrophages and nerve fibres in peritoneal endometriosis. Hum Reprod. (2009) 24:835–41. doi: 10.1093/humrep/den483, PMID: 19136478

[23] Greaves E Temp J Esnal-Zufiurre A Mechsner S Horne AW Saunders PTK. Estradiol is a critical mediator of macrophage-nerve cross talk in peritoneal endometriosis. Am J Pathol. (2015) 185:2286–97. doi: 10.1016/j.ajpath.2015.04.012, PMID: 26073038 PMC4530129

[24] Zaninelli TH Fattori V Heintz OK Wright KR Bennallack PR Sim D. Targeting NGF but not VEGFR1 or BDNF signaling reduces endometriosis-associated pain in mice. J Adv Res. (2024) 73:593–605. doi: 10.1016/J.JARE.2024.08.017, PMID: 39142441 PMC12225929

[25] Liang Y Xie H Wu J Liu D Yao S. Villainous role of estrogen in macrophage-nerve interaction in endometriosis. Reprod Biol Endocrinol. (2018) 16:1–11. doi: 10.1186/s12958-018-0441-z, PMID: 30518376 PMC6282253

[26] Duric V McCarson KE. Persistent pain produces stress-like alterations in hippocampal neurogenesis and gene expression. J Pain. (2006) 7:544–55. doi: 10.1016/j.jpain.2006.01.458, PMID: 16885011

[27] Mamillapalli R Gao X Taylor HS. Endometriosis alters anxiety, depression and pain perception as well as brain electrophysiology and gene expression in mice. Fertil Steril. (2017) 108:e43–4. doi: 10.1016/j.fertnstert.2017.07.142

[28] Bashir ST Redden CR Raj K Arcanjo RB Stasiak S Li Q. Endometriosis leads to central nervous system-wide glial activation in a mouse model of endometriosis. J Neuroinflamm. (2023) 20:1–18. doi: 10.1186/s12974-023-02713-0, PMID: 36879305 PMC9987089

[29] Maulitz L Nehls S Stickeler E Ignatov A Kupec T Henn AT. Psychological characteristics and structural brain changes in women with endometriosis and endometriosis-independent chronic pelvic pain. Hum Reprod. (2024) 39:2473–84. doi: 10.1093/humrep/deae207, PMID: 39241806

[30] As-Sanie S Kim J Schmidt-Wilcke T Sundgren PC Clauw DJ Napadow V. Functional connectivity is associated with altered brain chemistry in women with endometriosis-associated chronic pelvic pain. J Pain. (2016) 17:1–13. doi: 10.1016/j.jpain.2015.09.008, PMID: 26456676 PMC4698023

[31] Kasheh Farahani Z Taherianfard M Naderi MM Ferrero H. Assessing pain behavioral responses and neurotrophic factors in the dorsal root ganglion, serum and peritoneal fluid in rat models of endometriosis. J Family Reprod Health. (2020) 14(4):259–68. doi: 10.18502/JFRH.V14I4.5210, PMID: 34054998 PMC8144485

[32] Barcena De Arellano ML Arnold J Vercellino GF Chiantera V Ebert AD Schneider A. Influence of nerve growth factor in endometriosis-associated symptoms. Reprod Sci. (2011) 18:1202–10. doi: 10.1177/1933719111410711, PMID: 21673280

[33] Anaf V Simon P El Nakadi I Fayt I Simonart T Buxant F. Hyperalgesia, nerve infiltration and nerve growth factor expression in deep adenomyotic nodules, peritoneal and ovarian endometriosis. Hum Reprod. (2002) 17:1895–900. doi: 10.1093/humrep/17.7.1895, PMID: 12093857

[34] Farahani ZK Taherianfard M Naderi MM Ferrero H. Possible therapeutic effect of royal jelly on endometriotic lesion size, pain sensitivity, and neurotrophic factors in a rat model of endometriosis. Physiol Rep. (2021) 9:e15117. doi: 10.14814/phy2.15117, PMID: 34806344 PMC8606856

[35] Lian YL Cheng MJ Zhang XX Wang L. Elevated expression of transient receptor potential vanilloid type 1 in dorsal root ganglia of rats with endometriosis. Mol Med Rep. (2017) 16(2):1920–6. doi: 10.3892/mmr.2017.6783, PMID: 28627595 PMC5561994

[36] Greaves E Grieve K Horne AW Saunders PTK. Elevated peritoneal expression and estrogen regulation of nociceptive ion channels in endometriosis. J Clin Endocrinol Metab. (2014) 99:E1738–43. doi: 10.1210/jc.2014-2282, PMID: 25029427 PMC4207935

[37] Krisztina P Noémi B Péter M Béla K Zsuzsanna H. Presence and upregulation of Transient Receptor Potential Vanilloid 1 (TRPV1) and Ankyrin 1 (TRPA1) in translational rat endometriosis model. Bull Med Sci. (2019) 92:15–26. doi: 10.2478/orvtudert-2019-0011

[38] Castro J Maddern J Chow CY Tran P Vetter I King GF. The voltage-gated sodium channel NaV1.7 underlies endometriosis-associated chronic pelvic pain. J Neurochem (2024) 168:3760–76. doi: 10.1111/jnc.15795, PMID: 36840383

[39] Ding S Yu Q Wang J Zhu L Li T Guo X. Activation of ATF3/AP-1 signaling pathway is required for P2X3-induced endometriosis pain. Hum Reprod. (2020) 35:1130–44. doi: 10.1093/humrep/deaa061, PMID: 32303740

[40] Ding S Zhu L Tian Y Zhu T Huang X Zhang X. P2X3 receptor involvement in endometriosis pain via ERK signaling pathway. PloS One. (2017) 12:e0184647. doi: 10.1371/journal.pone.0184647, PMID: 28898282 PMC5595329

[41] Trapero C Martín-Satué M. Purinergic signaling in endometriosis-associated pain. Int J Mol Sci. (2020) 21:1–28. doi: 10.3390/ijms21228512, PMID: 33198179 PMC7697899

[42] Fattori V Zaninelli TH Rasquel-Oliveira FS Heintz OK Jain A Sun L. Nociceptor-to-macrophage communication through CGRP/RAMP1 signaling drives endometriosis-associated pain and lesion growth in mice. Sci Transl Med. (2024) 16(772):eadk8230. doi: 10.1126/SCITRANSLMED.ADK8230, PMID: 39504351

[43] Wang G Tokushige N Fraser IS. Nerve fibers and menstrual cycle in peritoneal endometriosis. Fertil Steril. (2011) 95:2772–4. doi: 10.1016/j.fertnstert.2011.01.150, PMID: 21334610

[44] Barcena de Arellano ML Arnold J Lang H Vercellino GF Chiantera V Schneider A. Evidence of neurotrophic events due to peritoneal endometriotic lesions. Cytokine. (2013) 62:253–61. doi: 10.1016/j.cyto.2013.03.003, PMID: 23545214

[45] Hirose M Kuroda Y Murata E. NGF/trkA signaling as a therapeutic target for pain. Pain Pract. (2016) 16:175–82. doi: 10.1111/papr.12342, PMID: 26452158

[46] Al-Jefout M Dezarnaulds G Cooper M Tokushige N Luscombe GM Markham R. Diagnosis of endometriosis by detection of nerve fibres in an endometrial biopsy: a double blind study. Hum Reprod. (2009) 24:3019–24. doi: 10.1093/humrep/dep275, PMID: 19690352

[47] Arnold J Barcena de Arellano ML Rüster C Vercellino GF Chiantera V Schneider A. Imbalance between sympathetic and sensory innervation in peritoneal endometriosis. Brain Behav Immun. (2012) 26:132–41. doi: 10.1016/j.bbi.2011.08.004, PMID: 21888965

[48] Hendriksen E van Bergeijk D Oosting RS Redegeld FA. Mast cells in neuroinflammation and brain disorders. Neurosci Biobehav Rev. (2017) 79:119–33. doi: 10.1016/j.neubiorev.2017.05.001, PMID: 28499503

[49] Forsythe P. Mast cells in neuroimmune interactions. Trends Neurosci. (2019) 42:43–55. doi: 10.1016/j.tins.2018.09.006, PMID: 30293752

[50] Morales JK Falanga YT Depcrynski A Fernando J Ryan JJ. Mast cell homeostasis and the JAK–STAT pathway. Genes Immun. (2010) 11:599. doi: 10.1038/gene.2010.35, PMID: 20535135 PMC3099592

[51] Urb M Sheppard DC. The role of mast cells in the defence against pathogens. PloS Pathog. (2012) 8(4):e1002619. doi: 10.1371/JOURNAL.PPAT.1002619, PMID: 22577358 PMC3343118

[52] Galli SJ Metz M Starkl P Marichal T Tsai M. Mast cells and IgE in defense against lethality of venoms: Possible “benefit” of allergy[. Allergo J Int. (2020) 29:46. doi: 10.1007/s40629-020-00118-6, PMID: 33224714 PMC7673288

[53] Ng MF. The role of mast cells in wound healing. Int Wound J. (2010) 7:55. doi: 10.1111/j.1742-481X.2009.00651.x, PMID: 20409251 PMC7951407

[54] Succar J Douaiher J Lancerotto L Li Q Yamaguchi R Younan G. The role of mouse mast cell proteases in the proliferative phase of wound healing in microdeformational wound therapy. Plast Reconstr Surg. (2014) 134:459–67. doi: 10.1097/PRS.0000000000000432, PMID: 24814421

[55] Weller K Foitzik K Paus R Syska W Maurer M Weller K. Mast cells are required for normal healing of skin wounds in mice. FASEB J. (2006) 20:2366–8. doi: 10.1096/fj.06-5837fje, PMID: 16966487

[56] Jin M Han T Yao Y Alessi AF Freeberg MA Inoki K. Glycolytic enzymes coalesce in G bodies under hypoxic stress. Cell Rep. (2017) 20:895–908. doi: 10.1016/j.celrep.2017.06.082, PMID: 28746874 PMC5586494

[57] Theoharides TC Valent P Akin C. Mast cells, mastocytosis, and related disorders. N Engl J Med. (2015) 373:163–72. doi: 10.1056/NEJMra1409760, PMID: 26154789

[58] Stasikowska-Kanicka O Danilewicz M Glłowacka A Wlłgrowska-Danilewicz M. Mast cells and eosinophils are involved in activation of ulcerative colitis. Adv Med Sci. (2012) 57:230–6. doi: 10.2478/v10039-012-0029-3, PMID: 22968338

[59] Toms R Weiner HL Johnson D. Identification of IgE-positive cells and mast cells in frozen sections of multiple sclerosis brains. J Neuroimmunol. (1990) 30:169–77. doi: 10.1016/0165-5728(90)90101-R, PMID: 2229408

[60] Mukai K Tsai M Saito H Galli SJ. Mast cells as sources of cytokines, chemokines and growth factors. Immunol Rev. (2018) 282:121. doi: 10.1111/imr.12634, PMID: 29431212 PMC5813811

[61] Solimando AG Desantis V Ribatti D. Mast cells and interleukins. Int J Mol Sci. (2022) 23:14004. doi: 10.3390/ijms232214004, PMID: 36430483 PMC9697830

[62] Chatterjea D Martinov T. Mast cells: versatile gatekeepers of pain. Mol Immunol. (2014) 63:38. doi: 10.1016/j.molimm.2014.03.001, PMID: 24666768 PMC4171343

[63] Mai L Liu Q Huang F He H Fan W. Involvement of mast cells in the pathophysiology of pain. Front Cell Neurosci. (2021) 15:665066. doi: 10.3389/fncel.2021.665066, PMID: 34177465 PMC8222580

[64] Kempuraj D Mentor S Thangavel R Ahmed ME Selvakumar GP Raikwar SP. Mast cells in stress, pain, blood-brain barrier, neuroinflammation and alzheimer’s disease. Front Cell Neurosci. (2019) 13:1–11. doi: 10.3389/fncel.2019.00054, PMID: 30837843 PMC6389675

[65] Cunha FQ Lorenzetti BB Poole S Ferreira SH. Interleukin-8 as a mediator of sympathetic pain. Br J Pharmacol. (1991) 104:765–7. doi: 10.1111/j.1476-5381.1991.tb12502.x, PMID: 1797337 PMC1908235

[66] Borelli V Martinelli M Luppi S Vita F Romano F Fanfani F. Mast cells in peritoneal fluid from women with endometriosis and their possible role in modulating sperm function. Front Physiol. (2020) 10:1543. doi: 10.3389/FPHYS.2019.01543, PMID: 31998139 PMC6964357

[67] McCallion A Nasirzadeh Y Lingegowda H Miller JE Khalaj K Ahn SH. Estrogen mediates inflammatory role of mast cells in endometriosis pathophysiology. Front Immunol. (2022) 13:961599. doi: 10.3389/FIMMU.2022.961599, PMID: 36016927 PMC9396281

[68] Paula R Oliani AH Vaz-Oliani DCM D’Ávila SCGP Oliani SM Gil CD. The intricate role of mast cell proteases and the annexin A1-FPR1 system in abdominal wall endometriosis. J Mol Histol. (2015) 46:33–43. doi: 10.1007/s10735-014-9595-y, PMID: 25201101

[69] Golinska M Rycerz A Sobczak M Chrzanowski J Stawiski K Fendler W. Complement and coagulation cascade cross-talk in endometriosis and the potential of JAK inhibitors – a network meta-analysis. medRxiv. (2025) 16:1619434. doi: 10.1101/2025.03.25.25324597, PMID: 40698088 PMC12279843

[70] Li T Wang J Guo X Yu Q Ding S Xu X. Possible involvement of crosstalk between endometrial cells and mast cells in the development of endometriosis via CCL8/CCR1. Biomed Pharmacother. (2020) 129:110476. doi: 10.1016/j.biopha.2020.110476, PMID: 32768961

[71] Anaf V Chapron C El Nakadi I De Moor V Simonart T Noël JC. Pain, mast cells, and nerves in peritoneal, ovarian, and deep infiltrating endometriosis. Fertil Steril. (2006) 86:1336–43. doi: 10.1016/j.fertnstert.2006.03.057, PMID: 17007852

[72] Sbei S Moncrief T Limjunyawong N Zeng Y Green DP. PACAP activates MRGPRX2 on meningeal mast cells to drive migraine-like pain. Sci Rep. (2023) 13:12302. doi: 10.1038/s41598-023-39571-y, PMID: 37516794 PMC10387048

[73] Meixiong J Anderson M Limjunyawong N Sabbagh MF Hu E Mack MR. Activation of mast-cell-expressed mas-related G-protein-coupled receptors drives non-histaminergic itch. Immunity. (2019) 50:1163–1171.e5. doi: 10.1016/j.immuni.2019.03.013, PMID: 31027996 PMC6531358

[74] Kim B Wang Z Meng X Xie Z Hor CC zhang w. An interorgan neuroimmune circuit promotes visceral hypersensitivity. Res Sq [Preprint]. (2025) 17:rs.3.rs-6221928. doi: 10.21203/rs.3.rs-6221928/v1, PMID: 40166016 PMC11957215

[75] Decraecker L Cuende Estévez M Van Remoortel S Quan R Stakenborg N Wang Z. Characterisation of MRGPRX2 ^+^ mast cells in irritable bowel syndrome. Gut. (2025) 74:1068–77. doi: 10.1136/gutjnl-2024-334037, PMID: 39988359

[76] Tauber M Basso L Martin J Bostan L Pinto MM Thierry GR. Landscape of mast cell populations across organs in mice and humans. J Exp Med. (2023) 220(10):e20230570. doi: 10.1084/jem.20230570, PMID: 37462672 PMC10354537

[77] Hillmer EJ Zhang H Li HS Watowich SS. STAT3 signaling in immunity. Cytokine Growth Factor Rev. (2016) 31:1. doi: 10.1016/j.cytogfr.2016.05.001, PMID: 27185365 PMC5050093

[78] Villarino AV Kanno Y Ferdinand JR O’Shea JJ. Mechanisms of Jak/STAT signaling in immunity and disease. J Immunol. (2015) 194:21. doi: 10.4049/jimmunol.1401867, PMID: 25527793 PMC4524500

[79] Buchert M Burns CJ Ernst M. Targeting JAK kinase in solid tumors: emerging opportunities and challenges. Oncogene. (2016) 35:939–51. doi: 10.1038/onc.2015.150, PMID: 25982279

[80] Qin JJ Yan L Zhang J Zhang WD. STAT3 as a potential therapeutic target in triple negative breast cancer: a systematic review. J Exp Clin Cancer Res. (2019) 38(1):195. doi: 10.1186/S13046-019-1206-Z, PMID: 31088482 PMC6518732

[81] Kim JW Gautam J Kim JE Kim JA Kang KW. Inhibition of tumor growth and angiogenesis of tamoxifen-resistant breast cancer cells by ruxolitinib, a selective JAK2 inhibitor. Oncol Lett. (2019) 17:3981. doi: 10.3892/ol.2019.10059, PMID: 30930994 PMC6425385

[82] Banerjee S Biehl A Gadina M Hasni S Schwartz DM. JAK–STAT signaling as a target for inflammatory and autoimmune diseases: current and future prospects. Drugs. (2017) 77:521. doi: 10.1007/s40265-017-0701-9, PMID: 28255960 PMC7102286

[83] Sarapultsev A Gusev E Komelkova M Utepova I Luo S Hu D. JAK-STAT signaling in inflammation and stress-related diseases: implications for therapeutic interventions. Mol Biomed. (2023) 4:40. doi: 10.1186/s43556-023-00151-1, PMID: 37938494 PMC10632324

[84] Park Y Han SJ. Interferon signaling in the endometrium and in endometriosis. Biomolecules. (2022) 12:1554. doi: 10.3390/biom12111554, PMID: 36358904 PMC9687697

[85] Kim S Lee Y Koo JS. Differential expression of lipid metabolism-related proteins in different breast cancer subtypes. PloS One. (2015) 10:1–15. doi: 10.1371/journal.pone.0119473, PMID: 25751270 PMC4353724

[86] Bian Y Yuan L Yang X Weng L Zhang Y Bai H. SMURF1-mediated ubiquitylation of SHP-1 promotes cell proliferation and invasion of endometrial stromal cells in endometriosis. Ann Transl Med. (2021) 9:362. doi: 10.21037/atm-20-2897, PMID: 33842583 PMC8033391

[87] Matsuzaki S Pouly JL Canis M. Persistent activation of signal transducer and activator of transcription 3 via interleukin-6 trans-signaling is involved in fibrosis of endometriosis. Hum Reprod. (2022) 37:1489–504. doi: 10.1093/humrep/deac098, PMID: 35551394

[88] Tanaka Y Yasugi T Nagayama M Sato M Ei SI. JAK/STAT guarantees robust neural stem cell differentiation by shutting off biological noise. Sci Rep. (2018) 8:1–9. doi: 10.1038/s41598-018-30929-1, PMID: 30127451 PMC6102247

[89] Wang T Yuan W Liu Y Zhang Y Wang Z Zhou X. The role of the JAK-STAT pathway in neural stem cells, neural progenitor cells and reactive astrocytes after spinal cord injury. BioMed Rep. (2014) 3:141. doi: 10.3892/br.2014.401, PMID: 25798237 PMC4360852

[90] Nicolas CS Amici M Bortolotto ZA Doherty A Csaba Z Fafouri A. The role of JAK-STAT signaling within the CNS. JAKSTAT. (2013) 2:e22925. doi: 10.4161/jkst.22925, PMID: 24058789 PMC3670265

[91] Gómez-Nicola D Valle-Argos B Pallas-Bazarra N Nieto-Sampedro M. Interleukin-15 regulates proliferation and self-renewal of adult neural stem cells. Mol Biol Cell. (2011) 22:1960–70. doi: 10.1091/mbc.e11-01-0053, PMID: 21508317 PMC3113763

[92] Dominguez E Rivat C Pommier B Mauborgne A Pohl M. JAK/STAT3 pathway is activated in spinal cord microglia after peripheral nerve injury and contributes to neuropathic pain development in rat. J Neurochem. (2008) 107:50–60. doi: 10.1111/j.1471-4159.2008.05566.x, PMID: 18636982

[93] Tsuda M Kohro Y Yano T Tsujikawa T Kitano J Tozaki-Saitoh H. JAK-STAT3 pathway regulates spinal astrocyte proliferation and neuropathic pain maintenance in rats. Brain. (2011) 134:1127–39. doi: 10.1093/brain/awr025, PMID: 21371995 PMC4571138

[94] Nicolas CS Peineau S Amici M Csaba Z Fafouri A Javalet C. The Jak/STAT pathway is involved in synaptic plasticity. Neuron (2012) 73(2):374–90. doi: 10.1016/j.neuron.2011.11.024, PMID: 22284190 PMC3268861

[95] Biggs CM Cordeiro-Santanach A Prykhozhij SV Deveau AP Lin Y Del Bel KL. Human JAK1 gain of function causes dysregulated myelopoeisis and severe allergic inflammation. JCI Insight. (2022) 7(24):e150849. doi: 10.1172/jci.insight.150849, PMID: 36546480 PMC9869972

[96] Minskaia E Maimaris J Jenkins P Albuquerque AS Hong Y Eleftheriou D. Autosomal dominant STAT6 gain of function causes severe atopy associated with lymphoma. J Clin Immunol. (2023) 43:1611–22. doi: 10.1007/s10875-023-01530-7, PMID: 37316763 PMC10499697

[97] Sharma M Lu HY Vaseghi-Shanjani M Del Bel KL Fornes O van der Lee R. Human germline heterozygous gain-of-function *STAT6* variants cause severe allergic disease. J Exp Med. (2023) 220(5):e20221755. doi: 10.1101/2022.04.25.22274265 36884218 PMC10037107

[98] Deng M Li Y Li Y Mao X Ke H Liang W. A novel STAT3 gain-of-function mutation in fatal infancy-onset interstitial lung disease. Front Immunol. (2022) 13:866638. doi: 10.3389/fimmu.2022.866638, PMID: 35677041 PMC9169891

[99] Cordeiro-Santanach A Prykhozhij SV Deveau AP Del Bel KL Turvey SE Berman J. A zebrafish JAK1-A634D gain-of-function model provides new insights into the pathogenesis of familial hypereosinophilia. Blood. (2018) 132:3060–0. doi: 10.1182/blood-2018-99-112124

[100] Milner JD Vogel TP Forbes L Ma CA Stray-Pedersen A Niemela JE. Early-onset lymphoproliferation and autoimmunity caused by germline STAT3 gain-of-function mutations. Blood. (2015) 125:591–9. doi: 10.1182/blood-2014-09-602763, PMID: 25359994 PMC4304103

[101] Agostinis C Balduit A Mangogna A Zito G Romano F Ricci G. Immunological basis of the endometriosis: the complement system as a potential therapeutic target. Front Immunol. (2021) 11:599117. doi: 10.3389/FIMMU.2020.599117, PMID: 33505394 PMC7829336

[102] Vallvé-Juanico J George AF Sen S Thomas R Shin MG Kushnoor D. Deep immunophenotyping reveals endometriosis is marked by dysregulation of the mononuclear phagocytic system in endometrium and peripheral blood. BMC Med. (2022) 20:1–19. doi: 10.1186/s12916-022-02359-4, PMID: 35421980 PMC9011995

[103] O’Shea JJ Kontzias A Yamaoka K Tanaka Y Laurence A. Janus kinase Inhibitors in autoimmune diseases. Ann Rheum Dis. (2013) 72:ii111. doi: 10.1136/annrheumdis-2012-202576, PMID: 23532440 PMC3616338

[104] Shawky AM Almalki FA Abdalla AN Abdelazeem AH Gouda AM. A comprehensive overview of globally approved JAK inhibitors. Pharmaceutics. (2022) 14:1001. doi: 10.3390/pharmaceutics14051001, PMID: 35631587 PMC9146299

[105] Huynh J Etemadi N Hollande F Ernst M Buchert M. The JAK/STAT3 axis: A comprehensive drug target for solid Malignancies. Semin Cancer Biol. (2017) 45:13–22. doi: 10.1016/j.semcancer.2017.06.001, PMID: 28647610

[106] Xin P Xu X Deng C Liu S Wang Y Zhou X. The role of JAK/STAT signaling pathway and its inhibitors in diseases. Int Immunopharmacol. (2020) 80:106210. doi: 10.1016/J.INTIMP.2020.106210, PMID: 31972425

[107] Farkas B Bessissow T Limdi JK Sethi-Arora K Kagramanova A Knyazev O. Real-world effectiveness and safety of selective JAK inhibitors in ulcerative colitis and crohn’s disease: A retrospective, multicentre study. J Clin Med. (2024) 13:7804. doi: 10.3390/jcm13247804, PMID: 39768726 PMC11728011

[108] Boyadzhieva Z Ruffer N Burmester G Pankow A Krusche M. Effectiveness and safety of JAK inhibitors in autoinflammatory diseases: A systematic review. Front Med (Lausanne). (2022) 9:930071. doi: 10.3389/fmed.2022.930071, PMID: 35833101 PMC9271622

[109] Kameda H. JAK inhibitors ∼ overview∼. Immunol Med. (2023) 46:108–11. doi: 10.1080/25785826.2023.2183594, PMID: 36850046

[110] Yadav P Wairkar S. Tofacitinib in focus: Fascinating voyage from conventional formulations to novel delivery systems. Int J Pharm. (2025) 671:125253. doi: 10.1016/j.ijpharm.2025.125253, PMID: 39842741

[111] Kim W Seo MK Kim YJ Choi SH Ku CR Kim S. Role of the suppressor of cytokine signaling-3 in the pathogenesis of Graves’ orbitopathy. Front Endocrinol (Lausanne). (2025) 16:1527275. doi: 10.3389/fendo.2025.1527275, PMID: 40104138 PMC11913680

[112] Gupta S Yamada E Nakamura H Perez P Pranzatelli TJF Dominick K. Inhibition of JAK-STAT pathway corrects salivary gland inflammation and interferon driven immune activation in Sjögren’s disease. Ann Rheum Dis. (2024) 83:1034. doi: 10.1136/ard-2023-224842, PMID: 38527764 PMC11250564

[113] Yang Y Dong Q Li R. Matrine induces the apoptosis of fibroblast-like synoviocytes derived from rats with collagen-induced arthritis by suppressing the activation of the JAK/STAT signaling pathway. Int J Mol Med. (2017) 39:307–16. doi: 10.3892/ijmm.2016.2843, PMID: 28035365 PMC5358712

[114] Suda Y Ikuta K Hayashi S Wada K Anjiki K Kamenaga T. Comparison of anti-inflammatory and anti-angiogenic effects of JAK inhibitors in IL-6 and TNFα-stimulated fibroblast-like synoviocytes derived from patients with RA. Sci Rep. (2025) 15:1–9. doi: 10.1038/s41598-025-94894-2, PMID: 40118969 PMC11928453

[115] Burchett JR; Dailey JM; Kee SA; Pryor DT; Kotha A; Kankaria RA. Targeting mast cells in allergic disease: current therapies and drug repurposing. Cells. (2022) 11:3031. doi: 10.3390/cells11193031, PMID: 36230993 PMC9564111

[116] Pinke KH Zorzella-Pezavento SFG de Campos Fraga-Silva TF Mimura LAN de Oliveira LRC Ishikawa LLW. Calming down mast cells with ketotifen: A potential strategy for multiple sclerosis therapy? Neurotherapeutics. (2019) 17:218. doi: 10.1007/s13311-019-00775-8, PMID: 31463682 PMC7007452

[117] Yanni JM Weimer LK Glaser RL Lang LS Robertson SM Spellman JM. Effect of lodoxamide on *in vitro* and *in vivo* conjunctival immediate hypersensitivity responses in rats. Int Arch Allergy Immunol. (1993) 101:102–6. doi: 10.1159/000236505, PMID: 7684628

[118] Bassler D Mitra AAD Ducharme FM Forster J Schwarzer G. Ketotifen alone or as additional medication for long-term control of asthma and wheeze in children. Cochrane Database Syst Rev. (2004) 2004(1):CD001384. doi: 10.1002/14651858.CD001384.PUB2, PMID: 14973969 PMC8406918

[119] Zhu L Lin K Zhang X Lin J. Sodium cromoglycate attenuates experimental endometriosis in rats by regulating mast cells. J Zhejiang Univ (Medical Sciences). (2022) 44:278–84. doi: 10.3785/j.issn.1008-9292.2015.05.07, PMID: 26350008 PMC10396824

[120] Minutello K Gupta V. Cromolyn Sodium. StatPearls (2024). Treasure Island (FL): StatPearls Publishing. Available online at: https://www.ncbi.nlm.nih.gov/books/NBK557473/. 29 Apr 2025., PMID: 32491405

[121] Gonzalez-Estrada A Reddy K Dimov V Eidelman F. Olopatadine hydrochloride ophthalmic solution for the treatment of allergic conjunctivitis. Expert Opin Pharmacother. (2017) 18:1137–43. doi: 10.1080/14656566.2017.1346085, PMID: 28656804

[122] Cao M Gao Y. Mast cell stabilizers: from pathogenic roles to targeting therapies. Front Immunol. (2024) 15:1418897. doi: 10.3389/fimmu.2024.1418897, PMID: 39148726 PMC11324444

